# Imitation of Gold

**Published:** 1893-05

**Authors:** 


### Imitation of Gold.

One of the most perfect forms is obtained by the following process: 100 parts by weight are taken of pure copper, 14 parts of tin or zinc, six of magnesia, 58 of sal ammoniac, 18 of quicklime, and nine of cream of tartar. The copper is melted, and to this are successively added the magnesia, sal ammoniac, quicklime, and cream of tartar, each by itself, in the form of powder; the whole is stirred for half an hour, the zinc or tin being added in small pieces, and stirring is resumed and continued till the whole is melted, the crucible being then covered and the mixture kept in a molten condition for the period of 35 minutes. After this the dross is carefully and entirely removed, and the metal is poured into moulds. The substance thus produced has a fine grain, is malleable, and does not easily tarnish.—*Ed. Brit. Jour. of Dental Science.*

